# AEG-1 is involved in hypoxia-induced autophagy and decreases chemosensitivity in T-cell lymphoma

**DOI:** 10.1186/s10020-018-0033-6

**Published:** 2018-07-09

**Authors:** Jiaqin Yan, Junhui Zhang, Xudong Zhang, Xin Li, Ling Li, Zhaoming Li, Renyin Chen, Lei Zhang, Jingjing Wu, Xinhua Wang, Zhenchang Sun, Xiaorui Fu, Yu Chang, Feifei Nan, Hui Yu, Xiaolong Wu, Xiaoyan Feng, Wencai Li, Mingzhi Zhang

**Affiliations:** 1 Department of Oncology, The First Affiliated Hospital, Zhengzhou University, No. 1 Jianshe East Road, Zhengzhou, Henan 450052 People’s Republic of China; 2 Department of Otorhinolaryngology, The Third Affiliated Hospital of Zhengzhou University, Zhengzhou, Henan 450052 People’s Republic of China; 3 Department of pathology, The First Affiliated Hospital, Zhengzhou University, Zhengzhou, Henan 450052 People’s Republic of China

**Keywords:** Astrocyte elevated gene-1 (AEG-1), T-cell non-Hodgkin’s lymphoma (T-NHL), Hypoxia, Autophagy, Chemosensitivity

## Abstract

**Background:**

This study was to examine the link between astrocyte elevated gene-1 (AEG-1) and hypoxia induced-chemoresistance in T-cell non-Hodgkin’s lymphoma (T-NHL), as well as the underlying molecular mechanisms.

**Methods:**

Expression of AEG-1, LC3-II, and Beclin-1 were initially examined in human T-NHL tissues (*n* = 30) and normal lymph node tissues (*n* = 16) using western blot, real-time PCR and immunohistochemistry. Western blot was also performed to analyze the expression of AEG-1, LC3-II, and Beclin-1 in T-NHL cells (Hut-78 and Jurkat cells) under normoxia and hypoxia. Additionally, the proliferation and apoptosis of Hut-78 cells exposed to different concentration of Adriamycin (ADM) in normoxia and hypoxia were evaluated by MTT and Annexin-V FITC/PI staining assay. Finally, the effects of AEG-1 on Hut-78 cells exposed to ADM in hypoxia were assessed by MTT and Annexin-V FITC/PI staining assay, and 3-MA (autophagy inhibitor) was further used to determine the underlying mechanism.

**Results:**

AEG-1, LC3-II and Beclin-1 expression were significantly increased in T-NHL tissues compared with normal tissues. Incubation of Hut-78 and Jurkat cells in hypoxia obviously increased AEG-1, LC3-II and Beclin-1 expression. Hypoxia induced proliferation and reduced apoptosis of Hut-78 cells exposed to ADM. AEG-1 overexpression further increased proliferation and decreased apoptosis of Hut-78 cells exposed to ADM in hypoxia. Moreover, overexpression of AEG-1 significantly inversed 3-MA induced-changes in cell proliferation and apoptosis of Hut-78 cells exposed to ADM in hypoxia.

**Conclusions:**

This study suggested that AEG-1 is associated with hypoxia-induced T-NHL chemoresistance via regulating autophagy, uncovering a novel target against hypoxia-induced T-NHL chemoresistance.

**Electronic supplementary material:**

The online version of this article (10.1186/s10020-018-0033-6) contains supplementary material, which is available to authorized users.

## Background

The lymphoma, a type of blood cancer, is roughly classified as Hodgkin’s lymphoma (HD) and non-Hodgkin’s lymphoma (NHL), and NHL represents the most common malignancy (Hadzipecova et al., 2007). T-cell lymphoma (T-NHL) accounts for approximately 15% of NHL in the United States (Tian et al., 2016). Currently, chemotherapy still remains the major choice for the treatment of T-NHL, especially at the advanced stages, but T-NHL is not that sensitive to conventional chemotherapy (R et al., 1987). These chemotherapy options ultimately yield poor outcomes in T-NHL patients, mainly resulted from the chemoresistance development of T-NHL. Actually, more than 90% of deaths from cancer are associated with drug resistance and metastasis (Ahmad et al., 2012).

Hypoxia is a common characteristic in solid tumors (Zhang et al., 2016a). Hypoxic environment triggers various adaptive responses in hepatocellular carcinoma (HCC) to survival in tough environment, and it provides a strong selective pressure for the survival of HCC, which results in the “survival of the fittest” and elimination of the inferior (Bogaerts et al., 2015; Zhang et al., 2016b). Reports also revealed that HCC cells in hypoxia are more resistant to chemotherapy than the cells growing in normoxia (Bogaerts et al., 2015; Zhang et al., 2016b; Lionel et al., 2012). Hypoxia in the tumor microenvironment is the major cause of drug resistance in cancer chemotherapy (Cosse & Michiels, 2008), but the mechanism by which hypoxia induces drug resistance in tumors is unclear. Several studies have shown that this process is mediated by autophagy. Song et al. (Song et al., 2009) found that autophagy was a protective way to participate in HCC chemotherapy resistance under hypoxic conditions, and chemotherapy induced-cell death in hypoxia was less than that in normoxia. They also observed that autophagy was significantly increased in hypoxia, and inhibition of autophagy by 3-MA or RNA interference increased cell death and improved drug resistance. In normoxia, antitumor drug 4-HPR resulted in cell death by inducing the apoptosis; while in hypoxia, 4-HPR induced autophagy, and 3-MA or chloroquine further enhanced apoptosis and reduced the survival of cells exposed to 4-HPR, suggesting that autophagy can prevent tumor cell death and may induce hypoxia-induced drug resistance to 4-HPR (Liu et al., 2011; XW et al., 2010). These studies fully demonstrate that autophagy is involved in the process of resistance induced by hypoxia.

Astrocyte elevated gene-1 (AEG-1) was initially cloned as neuropathology related gene in primary human embryos astrocytes in 2002 (Kang, 2002). Several researches have demonstrated the important role of AEG-1 in the progression of different tumors, including proliferation, metastasis, chemoresistance, and angiogenesis (Chang et al., 2016; X M & KK, 2013). Autophagy can be reflected by monitoring the accumulation of autophagy marker LC3- II. Silencing AEG-1 in a variety of tumor cell lines reduced LC3- II accumulation and restored chemosensitivity (Bhutia et al., 2010; Zou et al., 2016; Xie & Zhong, 2016). Besides, hypoxia inducible factor (HIF-1α) promoted AEG-1 expression by binding to the AEG-1 promoter (Zhao et al., 2017). However, it is unclear whether AEG1 participates in the regulation of autophagy and chemoresistance induced by hypoxia in T-NHL.

## Methods

### Tissue samples

Patients who were diagnosed with T-NHL at The First Affiliated Hospital of Zhengzhou University were included in the study after obtaining their oral and written informed consent. The biopsy specimens of patients (*n* = 30) were prepared by the Department of Clinical Pathology for paraffin-embedded tumor tissue sections. The control group consisted of 16 samples of lymph node that were obtained from normal lymph nodes in the disused tissues after standard operations, and the candidates were excluded from all kinds of tumors. This study was reviewed and approved by the Ethics Committee of the Medical Faculty at the First Affiliated Hospital of Zhengzhou University (Scientific Research-2017-LW-73).

### Cell culture and treatment

T-NHL cell lines (Hut-78 and Jurkat) were obtained from the Cell Bank of Chinese Academy of Science (Shanghai, China). Cells were cultured in RPMI-1640 medium supplemented with 10% heat-inactivated FBS (fetal bovine serum), 50 U/ml penicillin and 50 U/ml streptomycin (Sigma-Aldrich, St. Louis, MO, USA) at 37 °C in a humidified atmosphere containing 5% CO2.

Hypoxia treatment was performed by placing the cells in a sealed chamber (Thermo Forma) filled with mixture gases of 1% O2, 5% CO2, and 94% N2.

### Plasmid construction and cell transfection

The pcDNA3.1 vector was purchased from Invitrogen (USA). PcDNA3.1-AEG-1, a plasmid containing AEG-1, was constructed by Invitrogen (USA). The plasmid constructs carrying siRNA against AEG-1 and HIF-1α were designed and constructed as previously described (Yan et al., 2012) . Hut-78 and Jurkat cells were seeded in six-well plates at a density of 1 × 10^6^ cells per well. Subsequently, the transfection was performed by Lipofectamine™ 2000 (Invitrogen, USA) according to the manufacturer’s instructions. The stable transfection cells were verified for RT-PCR and western blot analysis.

### Immunohistochemical assay

Standard immunoperoxidase procedures were used to visualize AEG-1 and LC3-II expression, as previously described (Yan et al., 2012). Briefly, paraffin sections were deparaffinized in xylene, followed by a graded series of alcohols (100, 95 and 75%) and re-hydrated in water followed by Tris-buffered saline. Following antigen retrieval, slides were incubated with 3% H_2_O_2_ to prevent endogenous peroxidase. Then slides were blocked with 5% normal serum and incubated with anti-AEG-1 and anti- LC3-II antibody. After washing, the tissue sections were treated with biotinylated anti-rabbit secondary antibody (Zymed Laboratories Inc., South San Francisco, CA, USA), followed by further incubation with streptavidin-horseradish peroxidase complex (Zymed). Tissue sections were then immersed in 3, 3′-diaminobenzidine and counterstained with 10% Mayer’s hematoxylin, dehydrated and mounted.

### RNA extraction, reverse transcription and real-time PCR

Total-RNA from cultured cells was extracted using the TRIzol reagent according to the manufacturer’s instructions. The cDNA synthesis was performed in accordance with the protocol of the Takara Reverse Transcription System for real-time PCR [Takara Biotechnology (Dalian) Co., Ltd., China] with 2 μg RNA and reverse transcription performed with random primers. Real-time PCR primers were designed according to http://www.ncbi.nlm.nih.gov. The sequences of the PCR primers used were as follows: AEG-1, forward 5’-CGGTACCCCGGCTGGGTGAT-3′ and reverse 5’-CTCCTCCG CTTTTTGCGGGC-3′; HIF-1α, forward 5’-GTCGGACAGCCTCACCAAACAGAG C-3’and reverse 5’-GTTAACTTGATCCAAAGCTCTGAG-3′; GAPDH, forward 5’-CGGAGTCAACGGATTTGGTCGTATTGG-3′ and reverse 5’-GCTCCTGGAAGA TGGTGATGGGATTTCC-3′. Real-time PCR analysis was carried out on a LightCycler real-time PCR instrument using SYBR Green I kit (Tiangen Biotech Co., Ltd., Beijing, China) according to the manufacturer’s instructions. Each reaction was carried out in triplicate. Data were analyzed using the 2^-ΔΔCt^ method as described elsewhere (Fan et al., 2005).

### Western blotting assay

Total proteins were extracted by lysing cells in buffer (50 mM Tris pH 7.4, 150 mM NaCl, 0.5% NP-40, 50 mM NaF, 1 mM Na_3_VO_4_, 1 mM phenylmethylsulfonyl fluoride, 25 mg/ml leupeptin and 25 mg/ml aprotinin). The lysates were cleared by centrifugation and the supernatants were collected. Proteins were extracted using the protein extraction kit following the manufacturer’s instructions. Protein concentration was determined using protein assay reagent (Bio-Rad, Hercules, CA, USA). Equal amounts of protein were separated on SDS-PAGE, transferred to PVDF membranes, incubated with antibodies against AEG-1, HIF-1α, LC3-I, LC3-II, Beclin-1, and GAPDH, followed by incubation with the secondary antibodies. The membrane was then washed three times and visualized with diaminobenzidine. Quantification of the proteins was detected with the ECL system (Pierce Biotechnology Inc., Rockford, IL, USA). Each value represents the mean of triple experiments, and is presented as the relative density of protein bands normalized to GAPDH.

### MTT cell viability assay

MTT assay was carried out as previously described (Yan et al., 2012). Cells were seeded in a 96-well plate at a concentration of 2.5 × 10^4^/ml (100 μl/well). Six parallel wells were assigned to each group. Then, 20 μl/well of 5 mg/ml MTT (3-(4,5-dimethylthiazol-2-yl)-2,5-diphenyltetrazolium bromide) was added at different time after seeding and were then incubated for another 4 h. The supernatant was removed and the product converted from MTT was dissolved by adding 150 μl/well dimethylsulfoxide (DMSO). The plate was gently shaken for 15 min at room temperature and an enzyme-linked immunosorbent assay reader was used to measure the absorbance of each well at 570 nm.

### Annexin V-FITC flow cytometric analysis

Annexin V-FITC apoptosis detection kit (BD Biosciences, San Jose, CA, USA) was adopted to detect early apoptosis, as previously described (Yan et al., 2012). Briefly, after culturing for 48 h, each group of cells was harvested, washed twice with pre-chilled PBS and resuspended in binding buffer (HEPES-NaOH 10 mM pH 7.4, 144 mM NaCl and 25 mM CaCl_2_) at a concentration of 1 × 10^6^ cells/ml. One hundred microliters of this solution (1 × 10^5^ cells) was mixed with 5 μl of Annexin V-FITC and 5 μl of PI (BD Biosciences) according to the manufacturer’s instructions. The mixed solution was gently vortexed and incubated in the dark at room temperature (25 °C) for 15 min. Four hundred microliters of 1X dilution buffer were added to each tube and cell apoptosis analysis was performed by flow cytometry (BD FACSCalibur) within 1 h. At least 10,000 events were recorded and represented as dot plots.

### Statistical analysis

The SPSS13.0 software (SPSS, Inc., Chicago, IL, USA) was used for all statistical analyses, and results are expressed as mean ± SEM. The comparison between two groups was evaluated by Student’s t test; the comparison between multiple groups was performed using one-way analysis of variance (ANOVA), followed by the Tukey’s test. Results were considered statistically significant at *P* < 0.05.

## Results

### AEG-1, LC3-II, Beclin-1, and HIF-1α are significantly up-regulated in T-NHL tissues

**Fig. 1:**
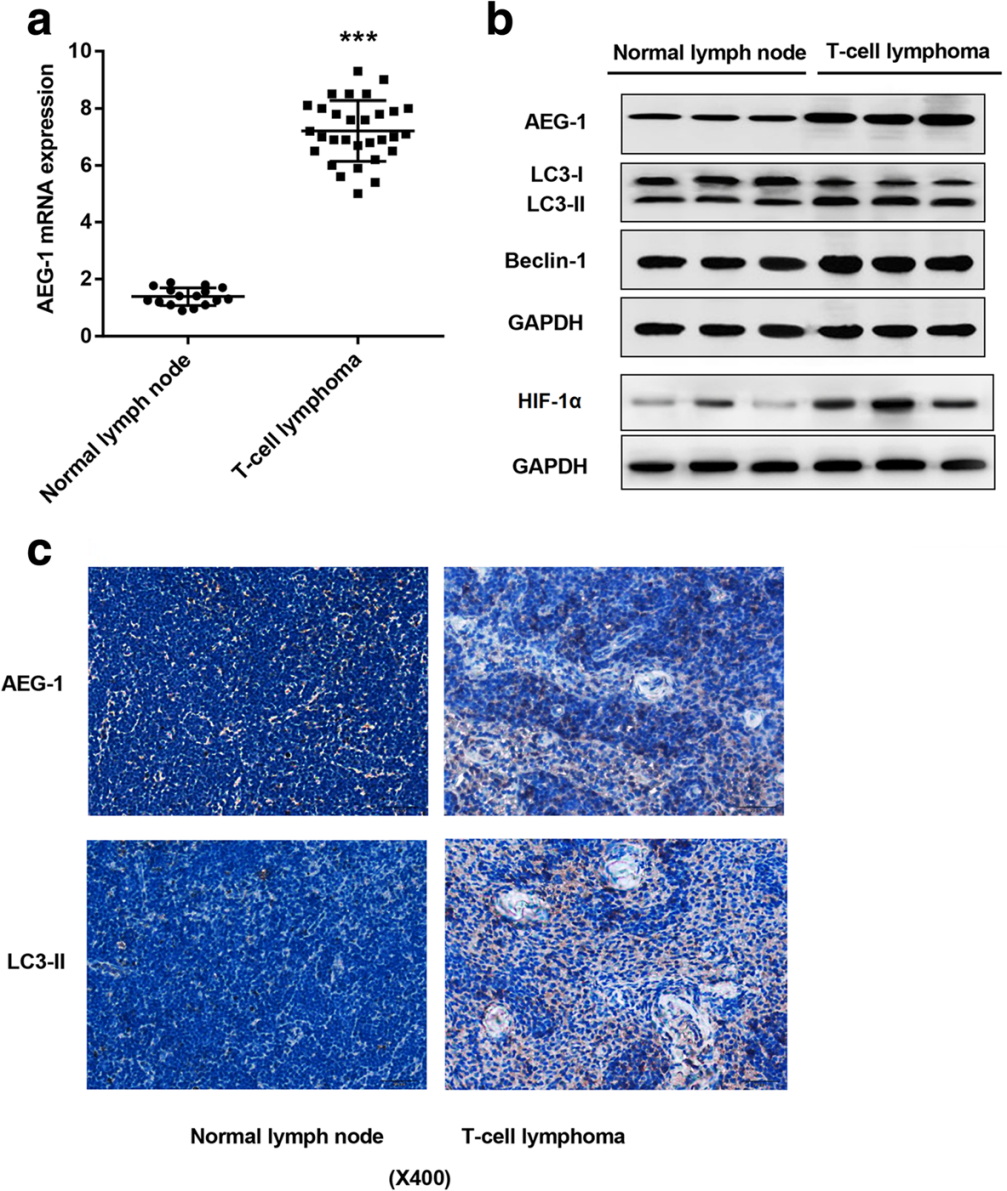
Relative expression of AEG-1, Beclin-1 and LC3-II in T-NHL tissues and normal lymphoid tissues. **a** Detection of AEG-1 in 30 T-NHL tissues and 16 normal lymphoid tissues using RT-PCR. **b** Expression of AEG-1, Beclin-1, LC3-I, LC3-II and HIF-1α were detected by western blot. **c** AEG-1 and LC3-II were detected by immunohistochemical assay. Bar = 20 μm. ^***^p < 0.001, T-NHL tissues vs. normal lymphoid tissues

To examine the expression of AEG-1 in T-NHL, tumor tissues (*n* = 30) and normal lymph node tissues (*n* = 16) were first employed and analyzed by RT-PCR and western blot. AEG-1 expression was significantly up-regulated in tumor tissues compared with normal tissues, both in mRNA (Fig. 1a) and protein levels (Fig. 1b). Western blot analysis also revealed the elevated levels of autophagy-related markers LC3-II and Beclin-1 in T-NHL tissues (Fig. 1b). Additionally, HIF-1α level was also elevated in T-NHL tissues (Fig. 1b). Immunohistochemical staining further confirmed high levels of AEG-1 and LC3-II in T-NHL tissues, which were rarely detected in normal tissues (Fig. 1c).

### Hypoxia triggers expression of AEG-1, LC3-II and Beclin-1 in T-NHL cells

**Fig. 2:**
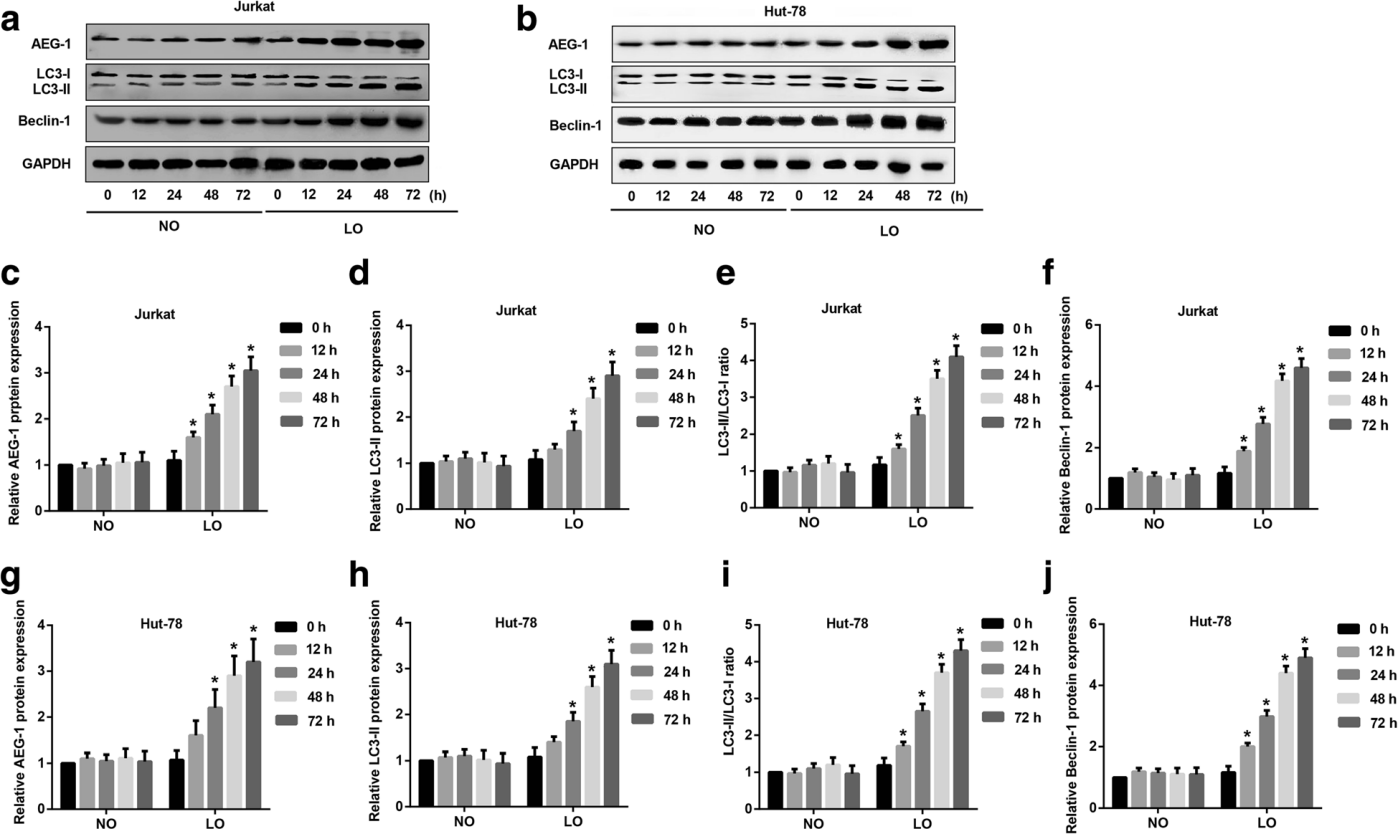
Effect of hypoxia on AEG-1 and autophagy markers in T-NHL cells. Hut-78 and Jurkat cells were incubated under normoxia or hypoxia for 0, 12, 24, 48 and 72 h before detection. **a** Expression of AEG-1, Beclin-1, LC3-I and LC3-II in Jurkat cells under normoxia and hypoxia environment via Western blot assays. **b** Expression of AEG-1, Beclin-1, LC3-I and LC3-II in Hut-78 cells under normoxia and hypoxia environment via Western blot assays. **c-f** Quantitative analysis of AEG-1, Beclin-1, LC3-I and LC3-II in Jurkat cells under normoxia and hypoxia. The expression of AEG-1 (*p* < 0.05), LC3-II (*p* < 0.05) and Beclin-1 (*p* < 0.05) was much higher in hypoxia than normoxia at 12, 24, 48 and 72 h. **g-j** Quantitative analysis of AEG-1, Beclin-1, LC3-I and LC3-II in Hut-78 cells under normoxia and hypoxia. The expression of AEG-1 (*p* < 0.05), LC3-II (*p* < 0.05) and Beclin-1 (*p* < 0.05) was much higher in hypoxia than normoxia at 12, 24, 48 and 72 h. NO: normoxia, NO: hypoxia. ^*^p < 0.05, NO vs. LO

To understand the effects of hypoxia on AEG-1 expression and autophagy in T-NHL, we detected the expression of AEG-1, LC3-II and Beclin-1 in hypoxia via western blot assay. Jurkat and Hut-78 cells were incubated in normoxia or hypoxia for 0, 12, 24, 48 and 72 h, respectively. The expression of AEG-1, LC3-II and Beclin-1 was much higher in hypoxia than normoxia at 12, 24, 48 and 72 h, both in Jurkat (Fig. 2a) and Hut-78 (Fig. 2b) cells. Quantitation analysis of western blot further confirmed up-regulated expression of AEG-1 (Fig. 2c), LC3-II (Fig. 2d) and Beclin-1 (Fig. 2e) as well as LC3-II/LC3-I ratio (Fig. 2f) in hypoxia, but not in normoxia in Jurkat cells. Similar results were also observed in Hut-78 cells (Fig. 2g-j). Additionally, the positive control for authophagy under starvation conditions further demonstrated that hypoxia induced authophagy in T-NHL cells through the detection of LC3-I, LC3-II and Beclin-1 expression (Additional file 1).

### Knocking down HIF-1α inhibits expression of AEG-1, LC3-II and Beclin-1 in T-NHL cells under hypoxia

**Fig. 3:**
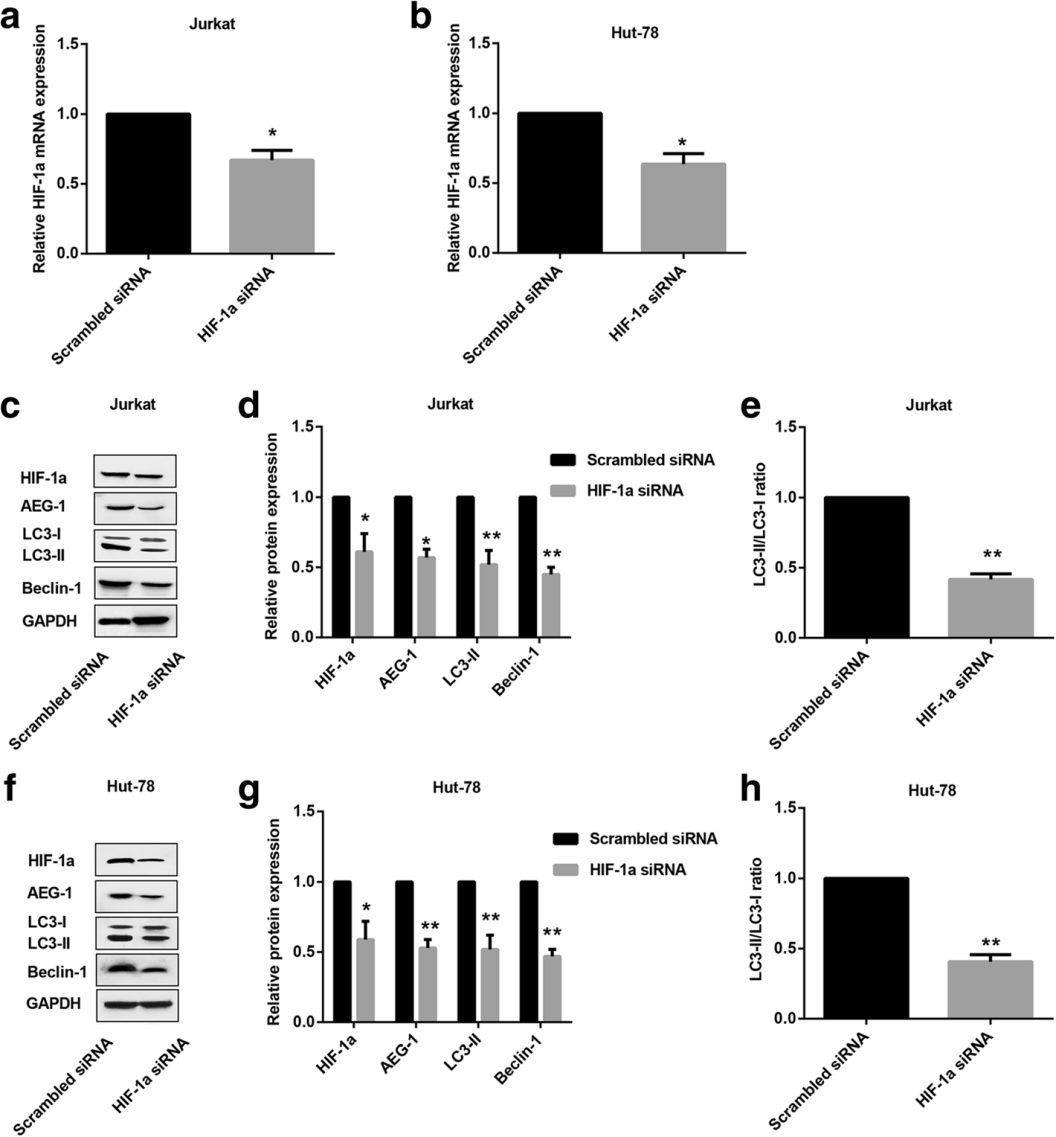
Effect of HIF-1α on AEG-1 and autophagy markers in T-NHL cells exposed to hypoxia. HIF-1α siRNA and the negative control (Scrambled siRNA) were transfected into Hut-78 and Jurkat cells, respectively. The mRNA expression of HIF-1α in Jurkat cells (**a**, *p* < 0.05) and Hut-78 cells (**b**, *p* < 0.05) were measured with RT-PCR. **c** Expression of HIF-1α, AEG-1, Beclin-1, LC3-I and LC3-II in Jurkat cells were detected by western blot. **d** Quantitative analysis of HIF-1α (*p* < 0.05), AEG-1 (*p* < 0.05), Beclin-1 (*p* < 0.01) and LC3-II (*p* < 0.01) in Jurkat cells. **e** Quantitative analysis of ratio of LC3-II/LC3-I (*p* < 0.01) in Jurkat cells. **f** Expression of HIF-1α, AEG-1, Beclin-1, LC3-I and LC3-II in Hut-78 cells were detected by western blot. **g** Quantitative analysis of HIF-1α (*p* < 0.05), AEG-1 (*p* < 0.01), Beclin-1 (*p* < 0.01) and LC3-II (*p* < 0.01) in Hut-78 cells. **h** Quantitative analysis of ratio of LC3-II/LC3-I (*p* < 0.01) in Hut-78 cells. ^*^p < 0.05, ^**^p < 0.01. HIF-1α siRNA vs.Scrambled siRNA

Transcriptional factor HIF-1α is a master regulator upon hypoxia, and it has been reported that HIF-1α promoted AEG-1 expression by binding to its promoter (Zhao et al., 2017). Here, to further elucidate the detailed role of HIF-1α in T-NHL under hypoxia, HIF-1α was first silenced in Jurkat (Fig. 3a) and Hut-78 (Fig. 3b) cells, and RT-PCR was performed to assess transfection efficiency. Moreover, western blot results revealed that AEG-1, LC3-II and Beclin-1 expression as well as LC3-II/LC3-I ratio were remarkably decreased in Jurkat cells transfected with HIF-1α siRNA under hypoxia (Fig. 3c-e). Similarly, under the hypoxic condition, Hut-78 cells transfected with HIF-1α siRNA exhibited the same trend (Fig. 3f-h).

### AEG-1 reduces chemosensitivity of Hut-78 cells under hypoxia

**Fig. 4:**
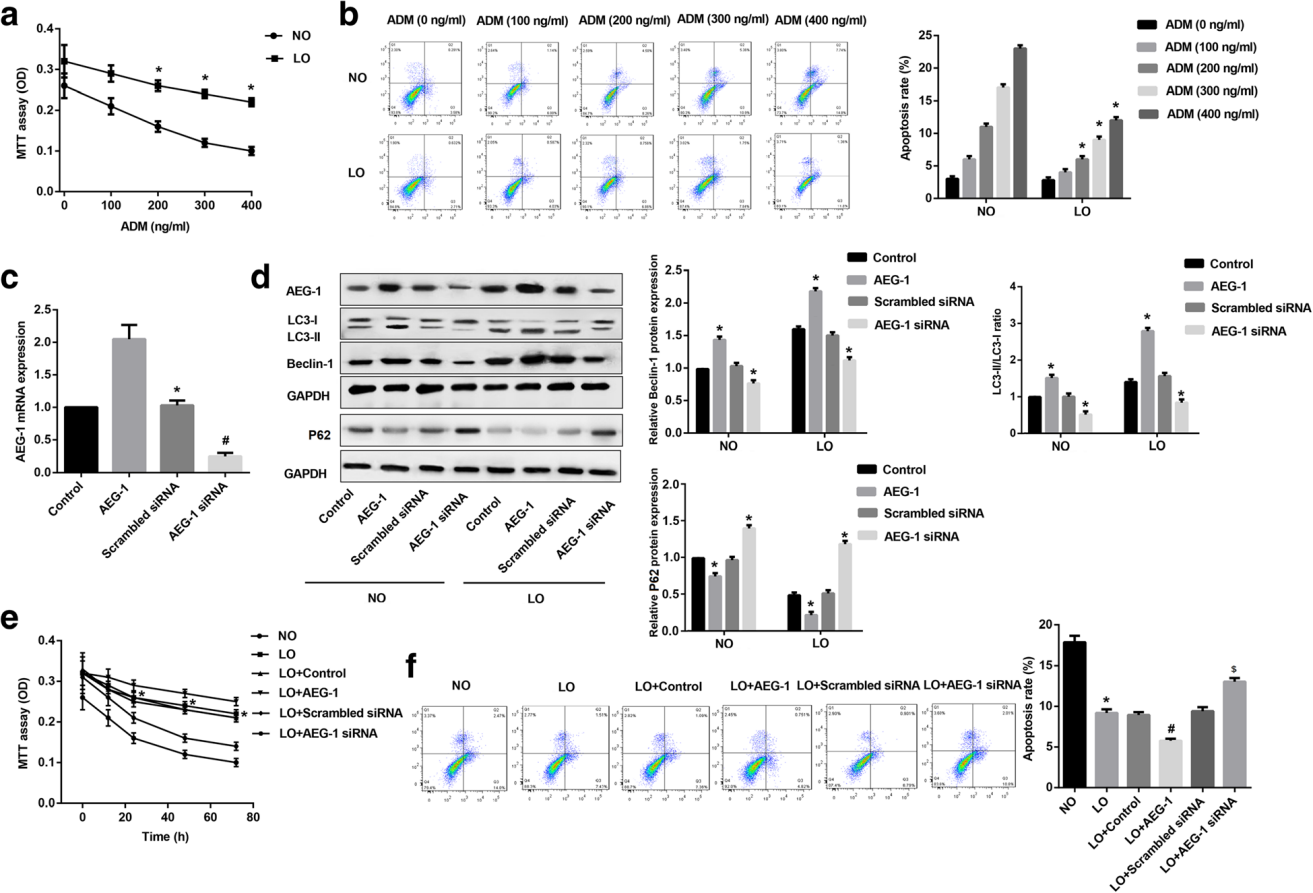
AEG-1 reduced chemosensitivity of Hut-78 cells in hypoxia. Hut-78 cells were cultured with different doses of ADM under normoxia or hypoxia for 24 h. Cell viability was detected by MTT assay (**a**, *p* < 0.05), and cell apoptosis was detected by Annexin-V FITC/PI double staining assay (**b**, *p* < 0.05). Hut-78 cells were transfected with pcDNA3.1-AEG-1 or AEG-1 siRNA. **c** The expression of AEG-1 (*p* < 0.05) was detected by RT-PCR. **d** Expression of AEG-1, Beclin-1, LC3-I, LC3-II, and P62 in Hut-78 cells under normoxia or hypoxia were detected by western blot. MTT assay (**e**) and Annexin-V FITC/PI double staining assay (**f**) were conducted in Hut-78 cells exposed to ADM under normoxia or hypoxia. ^*^p < 0.05 versus NO group. ^#^*p* < 0.05 versus LO + control group. ^$^*p* < 0.05 versus LO+ Scrambled siRNA group

Hut-78 cells were first treated with different doses of ADM under normoxia and hypoxia for 24 h. MTT assay revealed that ADM dose-dependently decreased cell viability both in normoxia and hypoxia, while cell viability in hypoxia was much higher than that in normoxia (Fig. 4a). In contrast, cell apoptosis was significantly increased in a dose-dependent manner both in normoxia and hypoxia, but the apoptosis of cells incubated in hypoxia was signally decreased compared with that in normoxia (Fig. 4b). These results indicated that hypoxia attenuated the response of Hut-78 cells to ADM. Then RT-PCR was performed to assess the transfection efficiency of pcDNA3.1-AEG-1 and AEG-1 siRNA in Hut-78 cells. AEG-1 expression was significantly increased in cells transfected with pcDNA3.1-AEG-1, but that was significantly decreased in cells transfected with AEG-1 siRNA (Fig. 4c). Besides, western blot revealed that AEG-1 overexpression markedly up-regulated Beclin-1 expression and LC3-II/LC3-I ratio, but those were significantly down-regulated in cells transfected with AEG-1 siRNA, both in normoxia and hypoxia (Fig. 4d). In contrast, p62 expression was markedly down-regulated in cells with AEG-1 overexpression, but AEG-1 siRNA significantly up-regulated p62 expression, both in normoxia and hypoxia (Fig. 4d). Especially, Beclin-1 expression and LC3-II/LC3-I ratio in hypoxia were prominently increased, while p62 expression in hypoxia were prominently decreased in comparison to normoxia (Fig. 4d). Further, under hypoxic conditions, AEG-1 overexpression signally enhanced the viability of Hut-78 cells following ADM treatment (Fig. 4e), while cell apoptosis was noteworthy reduced (Fig. 4f). These results indicated that AEG-1 blunted sensitivity of Hut-78 cells to ADM in hypoxia.

### AEG-1 reduces chemosensitivity of Hut-78 cells by promoting autophagy under hypoxia

**Fig. 5:**
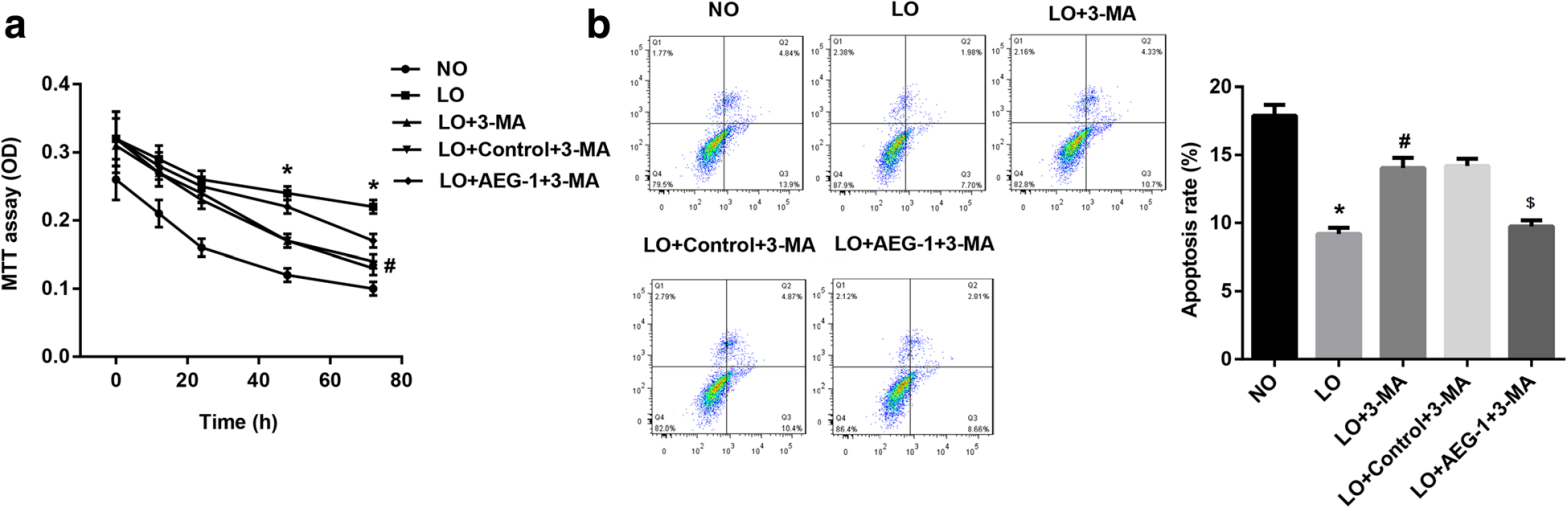
AEG-1 reduced chemosensitivity of Hut-78 cell by promoting autophagy. Hut-78 cells were cultured with 3-MA and ADM, and then transfected with pcDNA3.1-AEG-1. **a** Cell viability was detected by MTT assay. **b** Cell apoptosis was detected by Annexin-V FITC/PI double staining assay. ^*^p < 0.05 versus NO group. ^#^*p* < 0.05 versus LO group. ^$^*p* < 0.05 versus LO + Control+ 3-MA group

To illuminate the underlying mechanisms by which AEG-1 reduced the response of Hut-78 cells to ADM in hypoxia, 3-MA (10 mM, autophagy inhibitor) was employed (Nakanishi et al., 2016). MTT assay showed that inhibition of autophagy under hypoxia significantly decreased cell viability after ADM treatment, while AEG-1 partly improved the inhibition of cell viability by 3-MA (Fig. 5a). AEG-1 also significantly reversed the up-regulation of cell apoptosis induced by 3-MA in Hut-78 cells exposed to ADM under hypoxia (Fig. 5b).

## Discussion

Increasing evidence suggests that AEG-1 acts as an oncogene and is involved in many aspects of tumorigenesis, including protection from serum starvation-induced apoptosis, promoted tumor growth, angiogenesis and migration (Emdad et al., 2009; Emdad et al., 2007). High expression of AEG-1 has been reported in ovarian cancer tissues compared to normal ovarian tissues (Blanco et al., 2011). Besides, microarray analysis also confirmed that AEG-1 is associated with the regulation of chemoresistance (Meng et al., 2013). Actually, AEG-1 has been verified to be up-regulated in T-NHL and is associated with tumor growth in our previous study (Yan et al., 2012), but its effect on chemosensitivity in T-NHL is not understood.

The present study found that AEG-1 expression was remarkably increased in T-NHL specimens, which has been reported in our previous study (Yan et al., 2012). Besides, LC3-II and Beclin-1 were also remarkably increased in T-NHL specimens. In addition, AEG-1, LC3-II, and Beclin-1 were obviously induced in T-NHL cells in hypoxia. Hypoxia is also an important factor in promoting drug resistance. It can also be obtained that hypoxia blunted the response of Hut-78 cells to ADM, and overexpression of AEG-1 further enhanced the resistance of Hut-78 cells to ADM in hypoxia, as evidenced by cell viability and apoptosis assays. Above results indicated that AEG-1 was largely responsible for chemoresistance of Hut-78 cells in hypoxia, which was in accordance with the results observed in hepatocellular carcinoma (HCC) (Xie & Zhong, 2016).

In addition, we also proposed a mechanism by which AEG-1 enhanced chemoresistance of Hut-78 cells in hypoxia. A large amount of studies have demonstrated that autophagy plays a vital role in hypoxia-induced drug resistance (Liu et al., 2010; Ko et al., 2012; Rzymski et al., 2009). Thus, to illuminate the specific role of autophagy in chemoresistance of Hut-78 cells exposed to hypoxia, 3-MA (autophagic inhibitor) was selected. We found that inhibition of autophagy under hypoxia attenuated the cell viability and increased the apoptosis rate of Hut-78 cells, further, AEG-1 partially abolished the effect of 3-MA on the response of Hut-78 cells to ADM in hypoxia as revealed by MTT and apoptosis assays, indicating that AEG-1 reduced chemosensitivity of Hut-78 cells by inducing autophagy. It was reported that activation of autophagy inhibits tumor metastasis through the induction of HIF-1α (Indelicato et al., 2010). Previous studies have confirmed that hypoxia can induce autophagy through at least three pathways including activating transcription factor4, hypoxia-inducible factor1 and AMP-activated protein kinase (Liu et al., 2010; Rzymski et al., 2009; Kim et al., 2011). Actually, we also observed that the inhibition of HIF-1α significantly down-regulated AEG-1, LC3-II, Beclin-1 expression, and LC3-II/LC3-I ratio in T-NHL cells exposed to hypoxia. Unfortunately, the detail relationship between HIF-1α and AEG-1 in chemoresistance of Hut-78 cells exposed to hypoxia is not clear, which needs further investigation.

## Conclusion

In this paper, our data presents evidence that AEG-1, LC3-II, Beclin-1, and HIF-1α are significantly up-regulated in T-NHL tissues, and hypoxia triggers AEG-1, LC3-II and Beclin-1 expression in T-NHL cells (Hut-78 and Jurkat cells)*.* AEG-1 also reduces chemosensitivity of Hut-78 cells in hypoxia. Further, AEG-1 enhances chemoresistance of Hut-78 cells exposed to hypoxia by promoting autophagy. This study contributes to the target therapy against the drug resistance in T-NHL.

## Additional file

Additional file 1: Hut-78 and Jurkat cells were incubated under normoxia, hypoxia or starvation environment for 48 h before detection. a Western blot assays and quantitative analysis of AEG-1, Beclin-1, and LC3-II in Jurkat cells under normoxia, hypoxia or starvation environment. b Quantitative analysis of LC3-II/LC3-I ratio in Jurkat cells under normoxia, hypoxia or starvation environment. The expression of AEG-1 (p < 0.05), LC3-II (p < 0.05), Beclin-1 (p < 0.05) and LC3-II/LC3-I ratio (p<0.05) was much higher in hypoxia or starvation environment than normoxia in Jurkat cells. c Western blot assays and quantitative analysis of AEG-1, Beclin-1, and LC3-II in Hut-78 cells under normoxia, hypoxia or starvation environment. d Quantitative analysis of LC3-II/LC3-I ratio in Hut-78 cells under normoxia, hypoxia or starvation environment. The expression of AEG-1 (p < 0.05), LC3-II (p < 0.05), Beclin-1 (p < 0.05) and LC3-II/LC3-I ratio (p<0.05) was much higher in hypoxia or starvation environment than normoxia in Hut-78 cells. NO: normoxia, NO: hypoxia. *p < 0.05, NO vs. LO or starvation. (TIF 574 kb)

## Abbreviations

ADM: Adriamycin
AEG-1: Astrocyte elevated gene-1
ANOVA: One-way analysis of variance
DMSO: Dimethylsulfoxide
GAPDH: Glyceraldehyde-3-phosphate dehydrogenase
HCC: Hepatocellular carcinoma
HD: Hodgkin’s lymphoma
HIF-1α: Hypoxia inducible factor
NHL: Non-Hodgkin’s lymphoma
T-NHL: T-cell non-Hodgkin’s lymphoma
