# Supplementary figures and images for: AEG-1 is involved in hypoxia-induced autophagy and decreases chemosensitivity in T-cell lymphoma
Source: Mol Med. 2018 Jul 9;24:35. doi: 10.1186/s10020-018-0033-6 (PMC6038315; doi:10.1186/s10020-018-0033-6)

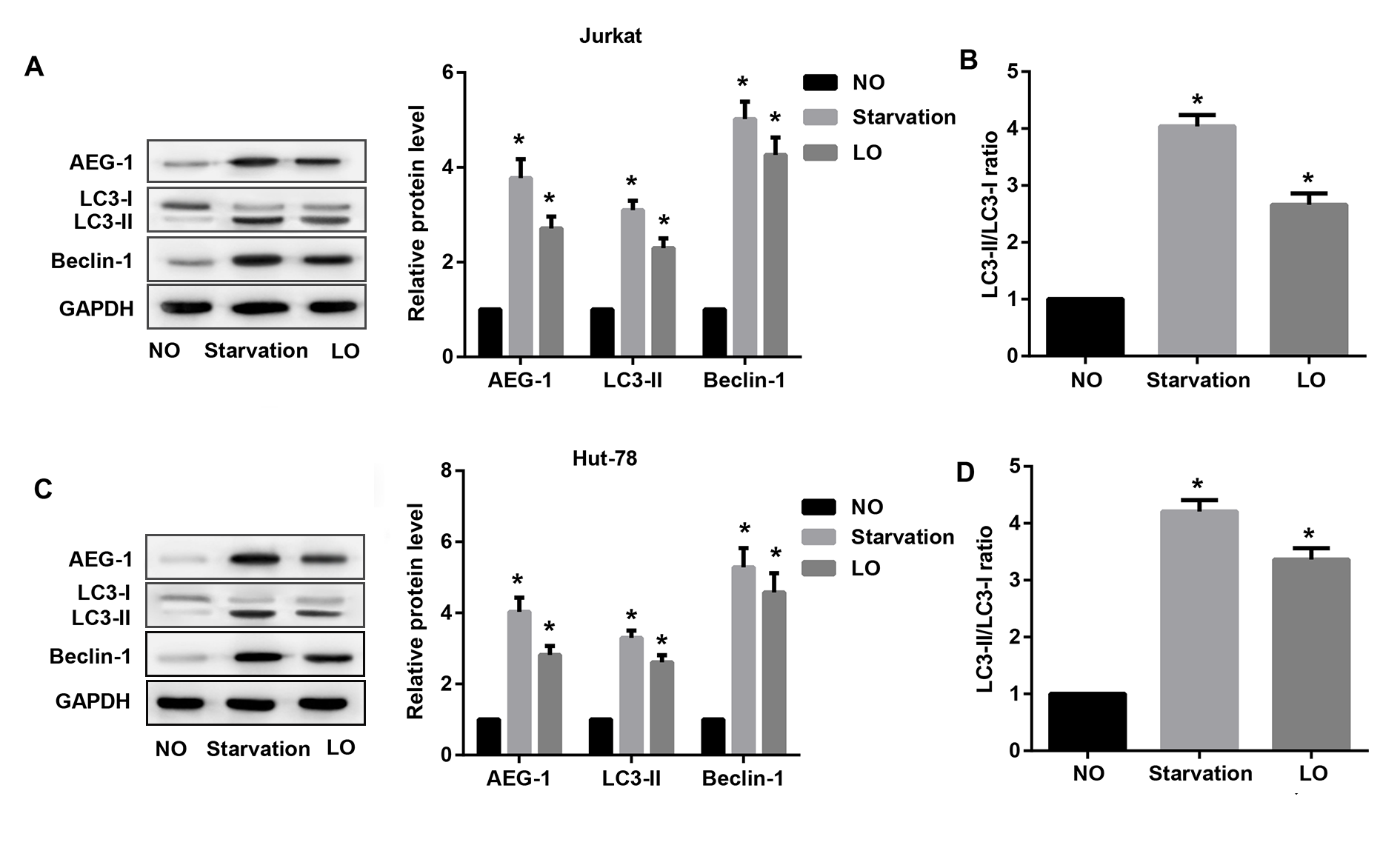

Supplement: Supplementary file 1 — Hut-78 and Jurkat cells were incubated under normoxia, hypoxia or starvation environment for 48 h before detection. a Western blot assays and quantitative analysis of AEG-1, Beclin-1, and LC3-II in Jurkat cells under normoxia, hypoxia or starvation environment. b Quantitative analysis of LC3-II/LC3-I ratio in Jurkat cells under normoxia, hypoxia or starvation environment. The expression of AEG-1 (p < 0.05), LC3-II (p < 0.05), Beclin-1 (p < 0.05) and LC3-II/LC3-I ratio (p<0.05) was much higher in hypoxia or starvation environment than normoxia in Jurkat cells. c Western blot assays and quantitative analysis of AEG-1, Beclin-1, and LC3-II in Hut-78 cells under normoxia, hypoxia or starvation environment. d Quantitative analysis of LC3-II/LC3-I ratio in Hut-78 cells under normoxia, hypoxia or starvation environment. The expression of AEG-1 (p < 0.05), LC3-II (p < 0.05), Beclin-1 (p < 0.05) and LC3-II/LC3-I ratio (p<0.05) was much higher in hypoxia or starvation environment than normoxia in Hut-78 cells. NO: normoxia, NO: hypoxia. *p < 0.05, NO vs. LO or starvation. (TIF 574 kb) [file 10020_2018_33_MOESM1_ESM.tif]
